# What Do People with Depression Want From EMA and Mood Monitoring Interventions? A Systematic Review and Qualitative Meta-Synthesis Assessing Usability, Acceptability, and Purpose

**DOI:** 10.1192/bjo.2025.10225

**Published:** 2025-06-20

**Authors:** Georgina Shajan, Daljit Purewal, Madiha Majid, Goldie Momoh, Laurence Astill Wright

**Affiliations:** 1 Institute of Mental Health, University of Nottingham, Nottingham, United Kingdom; 2 Coventry and Warwickshire Partnership NHS Trust, Coventry, United Kingdom; 3 Centre for Academic Mental Health, Population Health Sciences, University of Bristol, Bristol, United Kingdom

## Abstract

**Aims:** Advancements in digital technology have increased the potential for EMA to improve assessment efficiency through enabling real-time mood evaluation and raising the possibility of novel and technology informed interventions. The preferences and views of individuals with depression are crucial for the effectiveness of mood monitoring interventions or Ecological Momentary Assessment (EMA) as a data collection method. Concerns have been raised about the negative impact of frequent mood assessments. This is the first systematic review to our knowledge that assesses user experience of mood monitoring and EMA protocols. This systematic review and meta-synthesis evaluated the user experience of mood monitoring and EMA procedures, examining factors such as obstacles and facilitators for both people with depression and clinicians, potential adverse effects, and the intended goals of these methods.

**Methods:** A systematic review and meta-synthesis of qualitative studies on user and clinician experiences with mood monitoring and EMA for depression was conducted (PROSPERO: CRD42023396473). A search was performed across eight electronic databases. Qualitative studies exploring user perspectives on self-monitoring/EMA in people with depression were included. A meta-synthesis approach was applied to analyse the data, using first, second, and third-order constructs, following Noblit and Hare’s meta-ethnography framework. All qualitative studies were rated for risk of bias by two independent reviewers, and the results were verified for coherence by individuals with lived experience and psychiatrists.

**Results:** Fourteen studies met the inclusion criteria, from which seven themes emerged. These were: adverse effects, obstacles to mood tracking, enablers of mood tracking, the objective of mood monitoring, clinician-related challenges and concerns, clinician-driven recommendations and support, and desired features. All studies identified demonstrated a low risk of bias.

**Conclusion:** Many users reported a worsening of their mood and anxiety during EMA/mood monitoring. Users wanted to maintain control over their data and expressed a preference for a simple, intuitive, and passive data protocol. This review highlighted that personalisation should be a core feature of any future protocol development to maximise successful implementation and uptake of future protocol. These protocols should consider testing the incorporation of additional therapeutic elements to manage adverse effects as well as confirming these findings quantitatively. We present additional important concepts that are expected to enhance the user experience, engagement, retention, usability, and acceptance of EMA/mood monitoring protocols for individuals with depression.

